# Computer-Aided Drug Design of β-Secretase, γ-Secretase and Anti-Tau Inhibitors for the Discovery of Novel Alzheimer’s Therapeutics

**DOI:** 10.3390/ijms21030703

**Published:** 2020-01-21

**Authors:** Varnavas D. Mouchlis, Georgia Melagraki, Lefteris C. Zacharia, Antreas Afantitis

**Affiliations:** 1 Department of ChemoInformatics, NovaMechanics Ltd., Nicosia 1046, Cyprus; 2 Division of Physical Sciences & Applications, Hellenic Military Academy, Vari 16672, Greece; georgiamelagraki@gmail.com; 3 Department of Life and Health Sciences, University of Nicosia, Nicosia 1700, Cyprus; zacharia.l@unic.ac.cy

**Keywords:** Alzheimer’s disease, computer-aided drug design, β-secretase, γ-secretase, Tau, computational structure-based design, molecular docking, molecular dynamics, computational ligand-based design, QSAR, cheminformatics

## Abstract

Aging-associated neurodegenerative diseases, which are characterized by progressive neuronal death and synapses loss in human brain, are rapidly growing affecting millions of people globally. Alzheimer’s is the most common neurodegenerative disease and it can be caused by genetic and environmental risk factors. This review describes the amyloid-β and Tau hypotheses leading to amyloid plaques and neurofibrillary tangles, respectively which are the predominant pathways for the development of anti-Alzheimer’s small molecule inhibitors. The function and structure of the druggable targets of these two pathways including β-secretase, γ-secretase, and Tau are discussed in this review article. Computer-Aided Drug Design including computational structure-based design and ligand-based design have been employed successfully to develop inhibitors for biomolecular targets involved in Alzheimer’s. The application of computational molecular modeling for the discovery of small molecule inhibitors and modulators for β-secretase and γ-secretase is summarized. Examples of computational approaches employed for the development of anti-amyloid aggregation and anti-Tau phosphorylation, proteolysis and aggregation inhibitors are also reported.

## 1. Introduction

Alzheimer’s disease (AD) is a rapidly growing form of dementia affecting millions of people globally with devastating consequences to the patients and their families. According to the Alzheimer’s association, an estimated 5.8 million Americans of all ages are affected by AD including approximately 5.6 million people at the age of 65 and older and 0.2 million individuals under the age of 65 [1]. Health care and support of patients suffering from AD has a huge economic impact to families, health-care systems, and society. There is evidence of risk factors showing that lifestyle and other interventions contribute to reducing the future number of people to be diagnosed with AD or other forms of dementia [2]. However, the limited diagnostic methods and the absence of effective medical treatments for AD will continue to increase the burden on the society. Thus, understanding the molecular pathogenesis of AD is necessary for the development of improved diagnostic and therapeutic strategies [2].

AD is a Central Nervous System (CNS) disorder leading to neurodegeneration [3] which is linked to abnormal amyloid-β (Aβ) metabolism [4], Tau (tubulin-associated unit) hyperphosphorylation [5,6], oxidative stress [7,8], reactive glial [9], microglial changes [10], and other pathological abnormalities [3]. Even though research advances led to significant discoveries about the pathophysiology of AD, the molecular pathways involved in the development and progression of the disease are complex and not clearly understood [11]. Amyloid-β and Tau pathways leading to amyloid plaques and neurofibrillary tangles (NFTs), respectively, are the central point of research for the development of AD therapies. However, there is a high failure rate of drugs in clinical trials indicating the need for more intensive efforts regarding the development of new small molecule inhibitors [12]. Such compounds are excellent research tools in the hands of researchers working on developing novel therapeutic strategies for AD.

The development of small molecule drugs for AD is a challenging and laborious procedure due to the complexity of the molecular pathways involved in the progression of the disease [13]. Computer-Aided Drug Design (CADD) uses computer power, three-dimensional graphics, mathematics, and statistics to understand and predict the binding mode and energy of small molecule inhibitors with potential targets. The most common CADD techniques employed by medicinal chemists to help them rationalize the selection of hit compounds and to perform hit-to-lead optimization include computational structure-based design like molecular docking and dynamics and ligand-based design like quantitative structure-activity relationships (QSAR), descriptor-based QSAR, and pharmacophore mapping. The main molecular targets employed in the development of drugs against AD include β-secretase, γ-secretase, acetylcholinesterase, glycogen synthase kinase, muscarinic acetylcholine receptor and Tau protein [14]. In this review, the structure and function of druggable targets involved in the amyloid-β and Tau pathways are discussed. Examples for the application of CADD including computational structure-based design and ligand-based design approaches for the development of β-secretase, γ-secretase, anti-amyloid aggregation and anti-Tau phosphorylation, acetylation, and aggregation small molecule inhibitors and modulators are summarized as well.

## 2. The Amyloid-β Pathway and its Druggable Targets

**Figure 1:**
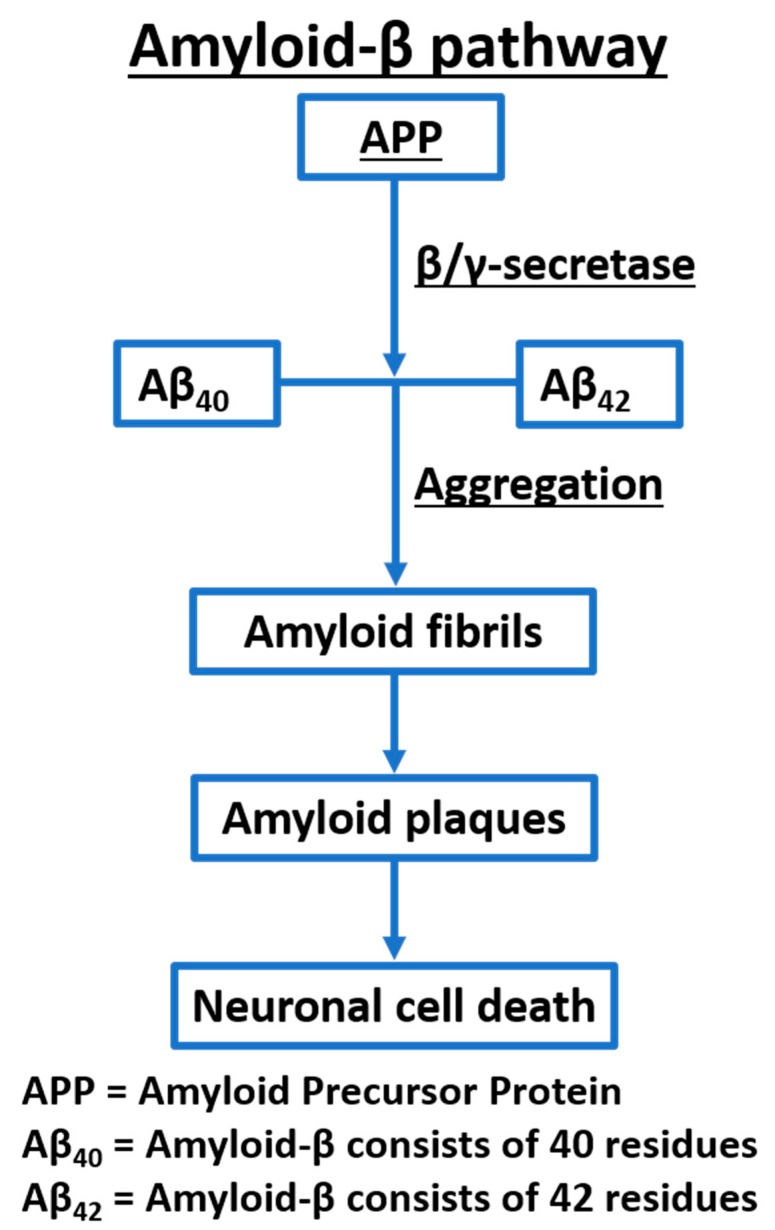
A simplified illustration of the amyloid-β pathway.

The amyloid cascade hypothesis, which explains the amyloid-β aggregation pathology, has been the predominant molecular pathway used for the development of AD drugs over the last 25 years (Figure 1). The enzymes β-secretase and γ-secretase sequentially cleave the amyloid precursor protein (APP) to produce the amyloid-β. Amyloid-β is a set of hydrophobic peptides consists of 40 (Aβ_40_) and 42 (Aβ_42_) residues [15]. Increased levels of the monomeric amyloid-β causes aggregation into amyloid fibrils which are responsible for the formation of plaques, causing neurotoxicity and induction of Tau pathology, leading to cell death and neurodegeneration [15]. Therefore, β-secretase and γ-secretase have been attractive molecular targets for the development of AD therapies.

### 2.1. Function and Structure of β-Secretase

**Figure 2:**
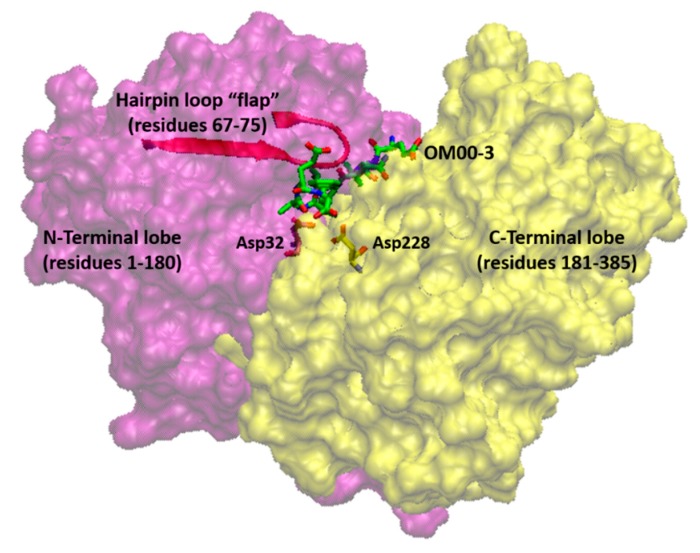
Three-dimensional structure of human BACE1 (PDB ID: 1FKN). The N-terminal lobe is colored in magenta and the C-terminal lobe is colored in yellow. The active site with the catalytic dyad of Asp32/Asp228 is located at the center of the two lobes with the co-crystallized inhibitor OM00-3.

The transmembrane enzyme β-secretase also known as β-site APP cleaving enzyme I (BACE1) is an aspartic protease, and the human enzyme was cloned in 1999 [16]. BACE1 has optimal enzymatic activity in an acidic pH [17], is mainly expressed in neurons, and its activity is higher in the Golgi apparatus, trans-Golgi network (TGN), secretory vesicles, and endosomes [16,18,19]. Electron microscopy revealed that BACE1 is subcellularly localized mostly within normal and dystrophic presynaptic terminals [20]. Chromosomal localization studies led to the identification of BACE2 which is a homologue of BACE1 [21]. Sequence alignment analysis showed that the amino acid sequences of human BACE1 and BACE2 gene coding regions are 45% identical and 75% homologous [22]. Numerus crystal structures of BACE1 have been resolved with and without inhibitor in its active site [23,24,25,26,27,28,29,30,31,32]. BACE1 contains an N-terminal protease domain, a connecting strand, a transmembrane region, and a cytosolic domain [23]. Its structure has an aspartic protease folding but its active site is more open compared to pepsin. The binding site of the substrate (also called “cleft”) is located between the N- and C-terminal lobes with the catalytic dyad of Asp32/Asp228 to be at the center of the cleft [28]. The cleft is covered by a β-hairpin loop (residues 67–75) also known as “flap” which is located at the N-terminal lobe and its conformational changes control substrate access (Figure 2).

**Figure 3:**
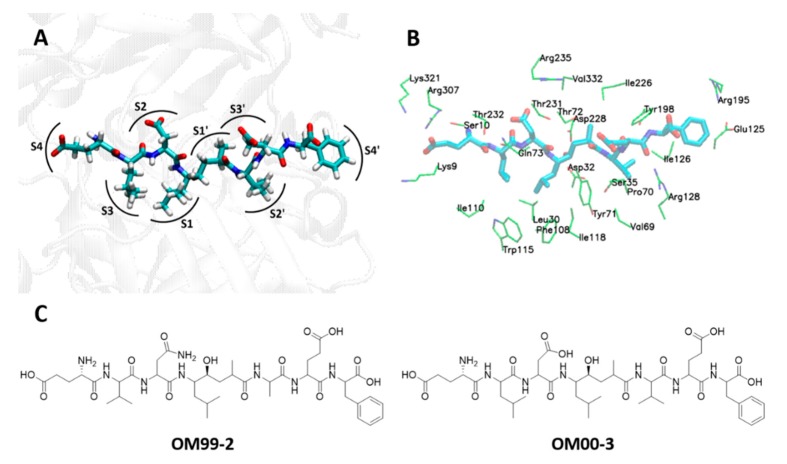
The active site of BACE1 and its inhibitors. (**A**) The subsites of BACE1 active site, (**B**) residues of BACE1 subsites interacting with OM00-3 inhibitor (PDB ID: 1M4H), and (**C**) 2D structures of OM99-2 and OM00-3 inhibitors.

The active site of BACE1 is large consisting of several subsites that are often called pockets (Figure 3A,B). Subsites S1 and S3 are in close proximity and they are hydrophobic containing residues Leu30, Phe108, Ile110, Ile118, and Trp115. The S2 and S4 pockets are solvent exposed with hydrophilic residues like Lys9, Ser10, Thr72, Gln73, Thr231, Thr232, Arg235, Arg307, and Lys321. These two pockets are also located near to each other. Exposed to the solvent is also the subsites S3′ and S4′ consisting of residues Pro70, Thr72, Glu125, Arg128, Agr195, and Trp197. Next to S4′ is located the S2′ subsite containing mostly hydrophobic and amphipathic residues like Ser35, Val69, Tyr71, Ile126, and Tyr198. The S1′ subsite is at the center of the active site and it contains the catalytic dyad of Asp32/Asp238 and two hydrophobic residues of Ile226 and Val332.

### 2.2. CADD for the Development of BACE1 Inhibitors

The development of BACE1 inhibitors was initiated with the discovery of OM99-2 (*Ki* = 1.6 nM) and OM00-3 (*Ki* = 0.32 nM) which are substrate-based inhibitors (Figure 3C) [33,34]. These two inhibitors were co-crystallized with BACE1 [23,35], and the elucidation of their binding mode with the active site of the enzyme was a critical point for the development of several BACE1 inhibitors [36]. Recent BACE1 inhibitors that were developed using CADD will be summarized in this section. In silico structure-based design was extensively employed in the development of BACE1 inhibitors including the discovery of peptides with a 5-fluoroorotyl moiety [37], 5,5′-disubstituted aminohydantoins [38], bicyclic iminopyrimidinones [39], iminopyrimidinones [40], cyclic sulfone hydroxyethylamines [41], imidazopyridines containing isoindoline-1,3-dione [42], iminochromene-2H-carboxamide derivatives containing different aminomethylene triazole [43], 2-substituted-thio-N-(4-substituted-thiazol/1H-imidazol-2-yl)acetamide derivatives [44], cyclopropane-based conformationally restricted analogues [45], 6-dimethylisoxazole-substituted biaryl aminothiazines [46], and other compounds [47]. In these studies, a combination of molecular docking, X-ray crystallography, synthesis, and in vitro testing was utilized to develop potent BACE1 inhibitors. *De novo* structure-based design led to the synthesis of compound libraries that were tested in vitro for identifying hit compounds including biphenylacetamide-derived BACE1 inhibitors [48]. Virtual screening revealed the impact of ligand protonation [49] and the importance of the protonation states of the catalytic dyad of Asp32/Asp228 in the discovery of hit compounds [50]. 

Ligand-based design is another CADD method for the development of small molecule inhibitors that is widely used when a receptor is not available. The abundance of BACE1 crystal structures allowed the development of hybrid structure-based virtual screening protocols, incorporating both structure-based and ligand-based design for identifying potential BACE1 inhibitors [51]. QSAR techniques were successful in developing structure-activity relationship models that are useful in predicting the binding affinity of potential BACE1 inhibitors [52]. Since BACE1 is highly flexible shifting its conformation from open to closed in the present of inhibitors, docking-based hybrid QSAR models demonstrated an efficient way to encompass receptor flexibility for predicting the inhibitory activity of structurally diverse sets of compounds [53]. A combination of molecular docking, molecular mechanics generalized Born surface area (MM-GBSA) calculations, virtual screening, and pharmacophore modeling led to the discovery of natural compounds as BACE1 inhibitors that were screened for anti-amyloidogenic activity using QSAR models [54]. Natural low molecular weight oligosaccharides that potentially inhibit BACE1 through interactions with the flap and catalytic dyad, were developed using virtual screening, molecular dynamics (MD) and 3D-QSAR [55]. A multi-target screening combining 2D-QSAR and molecular docking was successful in identifying hesperidin, a flavanone glycoside commonly found in citrus food items, that shows strong BACE1 inhibition, high Aβ aggregation inhibition, and moderate antioxidant activity [56]. QSAR classification models combining machine learning methods, model hybridizing strategies, backward elimination and visual analytics were developed for predicting putative BACE1 inhibitors [57]. A predictive self-organizing molecular field analysis (SOMFA) 3D-QSAR model for 5,5-disubstituted aminohydantoin was successful in studying the correlation of molecular properties and BACE1 inhibitory activities of these compounds [58]. Older ligand-based design studies are summarized in previously published reviews [59].

**Figure 4:**
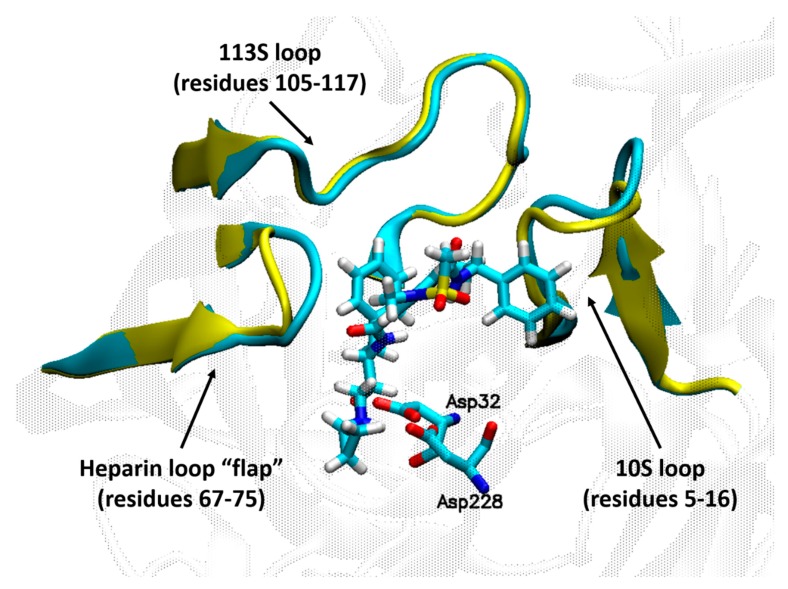
Flexibility of the loops surrounding the BACE1 active site revealed by the crystal structure of the enzyme with (cyan color, PDB ID: 3TPP) and without (yellow color, PDB ID: 3TPJ) a bound inhibitor.

Given the high flexibility of BACE1 that was demonstrated by the various crystal structures of the enzyme with or without co-crystalized inhibitors in its active site [28], BACE1 is an attractive target for MD studies. It has been reported that the flap, loop 10S and loop 113S have different conformations when BACE1 is crystallized with and without inhibitor in the active site (Figure 4). MD simulations revealed that an open conformation of the flap is often observed in the absence of an inhibitor in the active site of the enzyme, while interactions between the inhibitor and the flap drive the enzyme to adopt a closed conformation [28]. The protonation state of the catalytic dyad was determined using a combination of molecular docking and MD simulations [60,61]. Quantum mechanics/molecular mechanics (QM/MM) techniques were utilized to predict the binding energies of inhibitors for multiple therapeutic targets including BACE1 [62]. Multiple short MD simulations were coupled with MM-GBSA calculations to understand selectivity of BACE1 vs. BACE2 inhibitors [63]. Free energy perturbation (FEP) was successful in optimizing a novel series of amidine containing spirocyclic BACE1 inhibitors [64].

Some proteolytic enzymes contain additional binding pockets called exosites at locations distal to the catalytic active site where their hydrolytic enzymatic activity occurs [65,66]. Allosteric modulators that bind to exosites contribute to the stability or instability of the enzyme-substrate complex enhancing or inhibiting the enzymatic activity [67]. BACE1 is a challenging target for inhibitor development due the its open active site, which cannot be effectively inhibited by small molecules capable of penetrating the blood brain barrier. Therefore, intensive efforts to develop novel BACE1 modulators led to the discovery of exosites which are binding pockets distinct from the pockets of the catalytic active site [67,68,69,70]. Experimental evidence showed that it is possible to inhibit the enzymatic activity of BACE1 by targeting its exosites [69,71]. CADD studies for the development of BACE1 exosite modulators include molecular docking, MD simulations, and binding free energy calculations [72]. These studies showed that residues Glu255, Pro258, Phe261, Gly264-Ala272, Asp311-Ala313, Ser315, and Asp317-Tyr320 constitute an exosite binding pocket that interacts with a peptide modulator. A Molecular modeling study combined with mutagenesis gave structural and functional insights into the binding of the anti-BACE1 Fab fragment that recognizes the BACE1 exosite [73]. This study represents an in silico attempt to rationalize how BACE1 exosite modulators are capable to allosterically inhibit the enzyme. In particular, binding free energy calculations revealed that the anti-BACE1 Fab fragment recognizes the BACE1 functional epitope located within three loops consisting of residues 251–258, 270–273, and 311–317. Finally, MD simulations were combined with ab initio and density functional theory calculations to study the interactions of small peptides acting as BACE1 modulators [70]. In addition, the binding interactions of these peptides with exosite binding residues Glu163, Glu255, Lys256, Phe257, Pro258, Asp259, Gly260, Phe261, Trp262, Leu263, Gly264, Glu265, Gln266, Leu267, Val268, Cys269, Trp270, Gln271, Ala272, Gly273, Thr274, Asp311, Val312, Ala313, Thr314, Ser315, Gln316, Asp317, Asp318, Cys319, Tyr320, Lys321, and Phe322 were extensively determined within the framework of quantum and density functional theory of atoms [70].

### 2.3. Function and Structure of γ-Secretase

**Figure 5:**
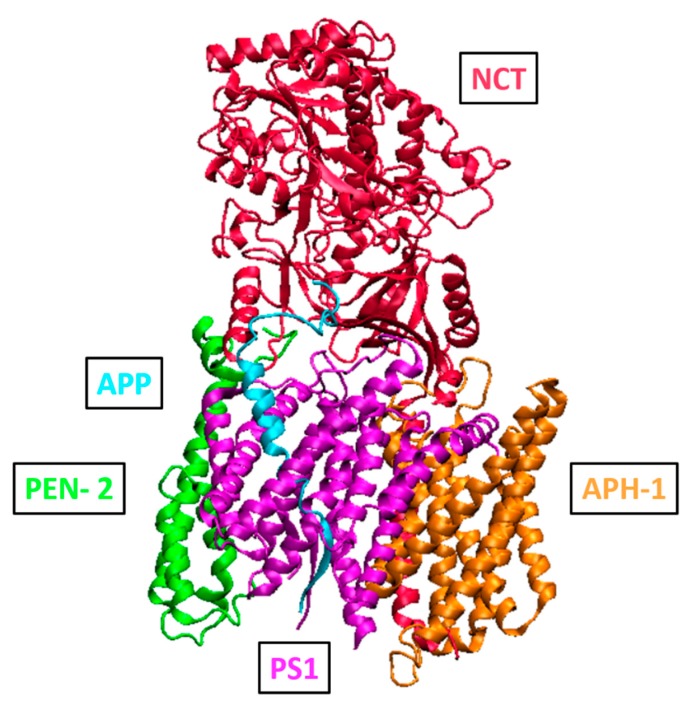
Three-dimensional structure of human γ-secretase complex (PDB ID: 6IYC).

The membrane-bound γ-secretase is a member of the intramembrane cleaving proteases (i-CLiP) which contains presenilin family of aspartyl proteases, rhomboid family of serine proteases, and zinc metalloprotease site-2 proteases [74,75]. It has been reported that γ-secretase possesses high enzymatic activity in a pellet prepared from rat brain enriched in Golgi, ER, endosomes, and synaptic vesicles [76]. Subcellular studies indicated that endosomes and synaptic structures are the main compartments of enzymatically active γ-secretase in rat brain [77]. Human γ-secretase is a high molecular weight complex containing four subunits (Figure 5): presenilin (PS), Nicastrin (NCT), anterior pharynx defective 1 (APH-1), and presenilin enhancer 2 (PEN-2) [78,79,80,81]. Several crystal structures of γ-secretase assembly have been resolved in the apo state [82,83], co-crystallized with Notch [84], with APP [85], and with inhibitors [82]. The aspartyl protease presenilin is the catalytic subunit of the complex existing in PS1 and PS2 isoforms [86]. Proteolysis of PS1 during the formation of γ-secretase complex results in an N-terminal fragment (NTF) and a C-terminal fragment (CTF) [87]. PEN-2 is required for maturation of the complex, while APH1 is responsible for its stability [88]. Finally, NTC was found to play an important role in the binding of APP [89]. In the crystal structure of γ-secretase assembly co-crystalized with DAPT, the inhibitor was found to interact with residues Ile143, Met146, Trp165, Leu166, Ser169, Met233, Phe283, Gly384, and Phe388, which constitute a binding pocket containing also the catalytic dyad of Asp257/Asp385 [82].

### 2.4. CADD for the Development of γ-Secretase Inhibitors

**Figure 6:**
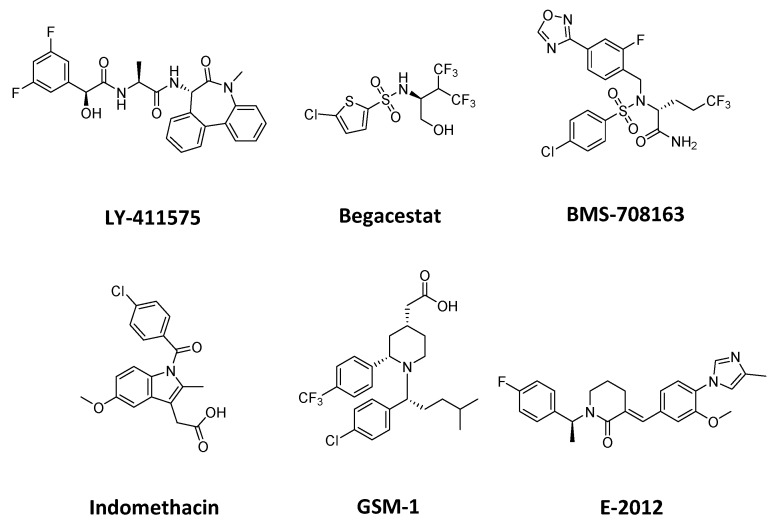
Two-dimensional structure of γ-secretase inhibitors (LY-411575, Begacestat, and BMS-708163) and γ-secretase modulators (Indomethacin, GSM-1, and E-2012).

Since γ-secretase was found to be a therapeutic target for AD, several γ-secretase inhibitors (GSIs) and γ-secretase modulators (GSMs) have been developed [90,91,92]. Examples of GSIs include LY-411575, Begacestat, and BMS-708163 (Figure 6) that were initially found to reduce the production of Aβ [92,93]. GSM examples are indomethacin, GSM-1, and E-2012 (Figure 6) that were found to modulate the production of Aβ_42_ compared to other Aβ peptides [91,94]. In this section, examples of CADD studies for the development of GSIs and GSMs are summarized. In silico screening of a drug library was performed using a combination of structure-based and ligand-based techniques including QSAR, pharmacophore models, and molecular docking [95]. Coarse-grained and druggability (also called mixed-probe, cosolvent-based, or mixMD) simulations were successful in identifying several “hot spots” for either orthosteric or allosteric inhibition of γ-secretase catalytic activity [96]. Multiscale MD simulations were employed to investigate substrate binding to γ-secretase and the role of substrate flexibility during the binding process [97]. MD simulations were combined with experimental techniques to map the binding site of BMS-708163 [98]. The molecular basis for Aβ peptides binding was studied with MD simulations and molecular docking indicating that the semi-open conformation of γ-secretase consistently shows the best binding mode for Aβ peptides, and it should be primarily targeted for the development of selective GSMs [99]. Atomistic microsecond-time scale MD simulations were combined with structural data, free energy calculations, and mutational data to identify the binding site of the C-terminal fragment of APP (C99) as well as the binding interactions of Aβ_49_, Aβ_46,_ and Aβ_43_ peptides [100]. The backbone dynamics of the substrate helix was investigated with hydrogen-deuterium exchange and MD simulation indicating that a diglycine hinge between the APP dimerization and cleavage domains may facilitate substrate positioning within PS1 active site [101]. The binding mode of a transition state analogue inhibitor (l-685, 458) was studied with MD simulation, docking, and free energy calculations revealing interactions of the inhibitor with the active site of γ-secretase and explaining the strongly reduced affinity of the epimer L682,679 [102]. The activity of known γ-secretase modulators was explored using neural network optimization and descriptors including hydrogen acceptor sites, the complexity of the drug measured by sum of degrees (S_D_), the desolvation energy penalty measured by the solvation energy of the drug, and the binding free energy to the protein revealing the importance of strong binding to multiple modulatory sites by favoring large complex molecules with a small dehydration penalty, many hydrogen-bond acceptor sites, and a favorable free binding energy [103]. MD simulations studies on γ-secretase have been also reported in other reviews [104].

### 2.5. CADD for the Development of Anti-Aβ Aggregation Inhibitors

Computational drug design was also employed for the development of anti-Aβ aggregation inhibitors. Examples include the development of an in silico model using Kohonen maps and counterpropagation artificial neural networks to explore the activity of 62 *N*-phenylanthranilic acids [105]. This model was successful in investigating various structural modifications that affect the biological activity to design novel structures using building block scaffolds and pharmacophore search by insertions, substitutions, and ring fusions of pharmacophoric substituents. A small library of peptide candidates for anti-Aβ aggregation inhibition were generated by employing molecular docking to computationally assessed mutations on an existing peptide (RGTFEGKF) inhibitor [106]. The final peptide library contained 300 peptides with up to four mutations per peptide that were rescored, and the three top scoring candidate inhibitors were further assessed with steered MD simulations showing higher binding free energies compared to the original peptide. The structural features associated with the high toxicity of Aβ_42_ were studied by utilizing straightforward extensive MD, steered MD, and replica exchange MD simulations [107]. In this study, the Aβ_42_ triple-β structure consisting of three β-sheet regions and two hydrophobic cores between these regions, demonstrated structural stability because of its complex residual interactions that stabilize the hydrophobic cores. A computational structure-based pharmacophore model based on docking poses of a peptide inhibitor (PGKLVYA) consisting of two hydrophobic, one hydrogen-bond donor, and one positive ionizable feature was employed as a 3D query in virtual screening to identify new hit Aβ aggregation inhibitors [108]. Selected hits were subsequently evaluated by toxicity prediction, molecular docking, and MD simulations to finally identify two hits (NSC35984 and NSC102747) as promising candidates for Aβ aggregation inhibition. Docking calculations and MD simulations revealed the binding mode of two d-amino acid pseudo-peptides that were designed to stop Aβ aggregation [109]. A substituted peptide inspired from MD simulations was studied using NMR and found to prevent mature fibril and β-sheet formation [110]. In the same study, ligand-based drug design led to the identification of a lead compound that has similar anti-Aβ aggregation properties as the peptide. The structural and physicochemical requirements for the potential Aβ aggregation inhibition were investigated on a set of 30 curcumin derivatives using docking, 2D-QSAR, HQSAR, and 3D QSAR [111]. The Aβ aggregation inhibitory activities of newly design molecules were predicted through inverse QSAR, and further screened using machine learning and toxicity prediction models to finally identify six lead compounds. A set of Aβ_42_ structures selected from clusters of conformations within an ensemble generated by MD simulations were used to perform fragment mapping calculations and to identify binding “hot spots” on the monomeric form of the Aβ_42_ peptide. The identified binding pockets exhibited a propensity to bind small molecules and they can be utilized for fragment-based drug design of anti-Aβ aggregation inhibitors [112]. Novel curcumin derivatives were proposed as Aβ aggregation inhibitors using docking calculations and ab initio fragment molecular orbital methods [113]. The results showed that curcumin derivatives bind strongly to the Aβ peptide. Finally, computational structure-based design was employed for the development of a series of peptide inhibitors for Aβ aggregation [114].

## 3. Therapeutic Strategies Targeting Tau in Tauopathies

**Figure 7:**
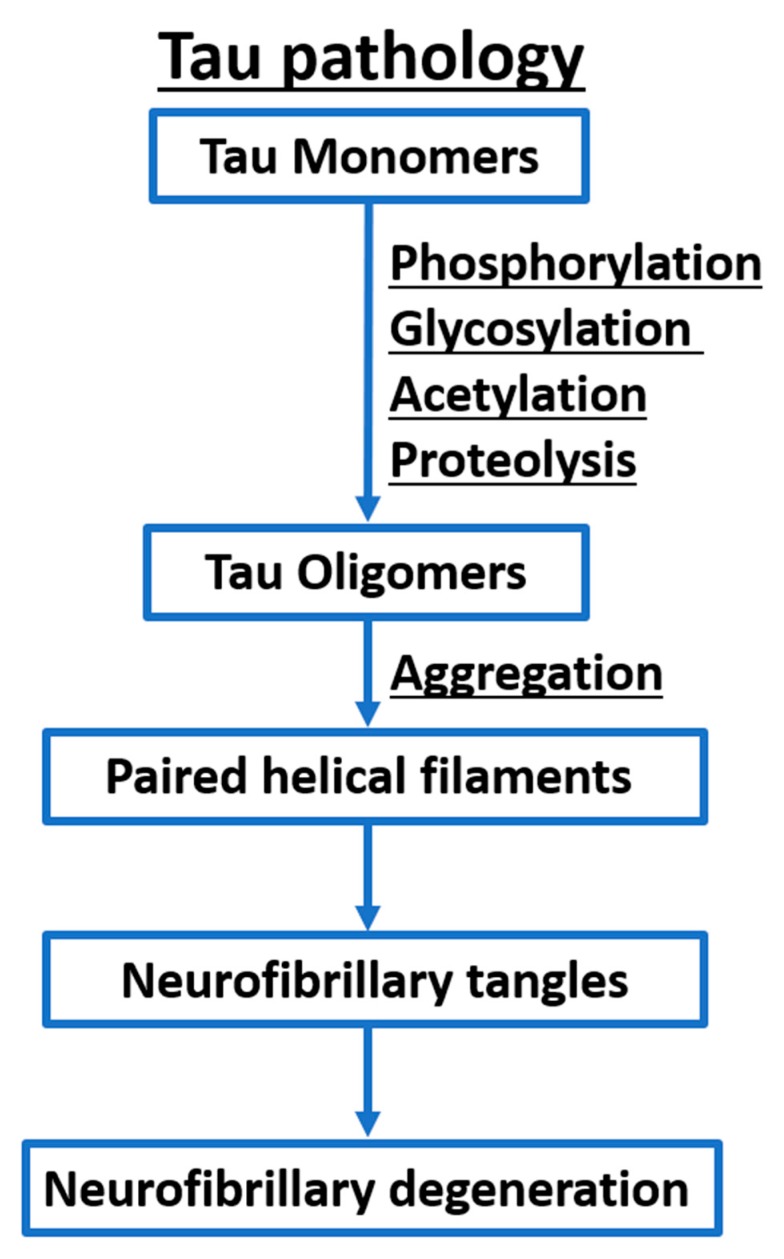
A simplified illustration of Tau pathology.

**Figure 8:**
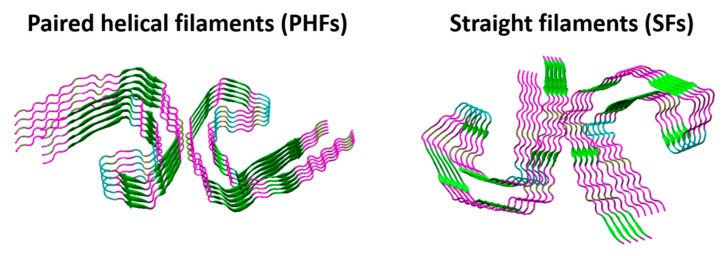
β-sheet structures of aggregated Tau that form paired helical filaments (PDB ID: 5O3L) and straight filaments (PDB ID: 5O3T).

In a number of neurodegenerative disorders including AD, monomers of soluble Tau accumulates in the cell and begins to form oligomers that self-assemble into aggregates in which Tau has a more complicated conformation [115,116]. The β-sheet structures of aggregated Tau form paired helical filaments (PHFs) and straight filaments (SFs), which assemble to intraneuronal fibrillar deposits known as NFTs leading to neurofibrillary degeneration (Figure 7 and Figure 8) [117]. Post-translational modifications including phosphorylation [118], glycosylation [119], acetylation [120], and truncation [121] differentiate pathological from normal Tau found in healthy brains. Phosphorylation sites include but not limited to Ser-Pro and Thr-Pro motives and it was reported that hyperphosphorylation affects the shape and the biological activity of Tau [122,123,124]. The most well studied kinases that phosphorylate Tau include glycogen synthase kinase-3β (GSK-3β), cyclin-dependent kinase 5 (Cdk5), casein kinase 1 (CK1), and protein kinase A (PKA) [125]. It is worth mentioning that the activity of protein phosphatase 2 (PP2A), which is responsible for more than 70% of dephosphorylated Tau at most of its phosphorylation sites, is significantly compromised in AD brains [126]. Multiple O-glycosylation sites of Ser or Thr have been experimentally confirmed for Tau including Thr123, Ser208, Ser400, and Ser409/Ser412/Ser413 [127,128,129]. Residues Lys280 and Lys274 were identified as acetylation sites associated with Tau pathology [120,130]. Acetylation of Tau was also identified at the KXGS motif in the microtubule-binding domain suggesting that this acetylation play a critical role in Tau aggregation and clearance [131]. Proteolytic cleavage at the C-terminus of Tau have also been associated to the pathogenesis of AD [121]. The main enzymes implicated in Tau proteolysis include caspases, calpains, cathepsins, and a thrombin-like protease.

### 3.1. Function and Structure of Tau

Tau was isolated from porcine brain extracts in 1975 [132], and it was purified and characterized in 1977 [133,134]. Tau is mainly expressed in axons of CNS neurons and can be also found in somatodendritic compartment of neurons, oligodendrocytes, and non-neural tissues [135,136]. The main function of Tau protein is to promote stability of microtubules along with other microtubule-associated proteins [5]. A second isoform of Tau was identified in 1988, which is identical to the first with the exception of an insert of 31 amino acids in the repeat region [137]. Sequencing studies revealed the existence of six human Tau isoforms ranging from 352 to 441 residues in length [138]. Two functional domains were identified in Tau including the N-terminal domain also called “projection domain” (residues 1–186) that does not bind to microtubules and the C-terminal microtubule-binding domain also called “assembly domain” (residues 187–430) because it promotes Tau self-assembly [139,140]. Tau is highly hydrophilic and does not adopt a compact folded structure which is typical to cytosolic proteins, but it is rather natively unfolded or intrinsically disorder and very flexible [140]. Model peptides for aggregation studies include fragments from isoforms K18 (isoform with 4 repeat domain) and K19 (isoform with 3 repeat domain) containing the assembly domain [117]. Hexapeptides PHF6 * (^275^VQIINK^280^) and PHF6 (^306^VQIVYK^311^) are located at the beginning of the second and third repeat region playing a central role in Tau aggregation [141]. Structures of PHF6* and PHF6 were solved using micro-electron diffraction (microED) [142,143]. The atomic structure of Tau (residues 254–290) revealed the interactions between actin filaments and microtubule-associated proteins [144]. The NMR structure of Tau (residues 267–312) showed that Tau adopts a unique conformation upon binding to microtubules [145]. Cryo-electron microscopy (Cryo-EM) was successfully used to solve the three-dimensional structure of PHFs and SFs from the brain of AD patients, which may be useful for designing Tau aggregation inhibitors (Figure 8) [146,147].

### 3.2. CADD for the Development of Anti-Tau Inhibitors

Anti-Tau therapeutic strategies for the development of small molecule inhibitors include inhibition of Tau phosphorylation, aggregation, proteolysis, and microtubules stabilization [5]. In this section, CADD techniques employed for the development of anti-Tau therapeutics are summarized. Molecular docking and enzymatic kinetics demonstrated that C-glycosylflavones selectively inhibited GSK3β-mediated Tau hyperphosphorylation acting as a substrate competitive inhibitor [148]. A group of marine indole alkaloids isolated from the marine tunicate Aplidium were identified as scaffolds for designing new anti-Tau hyperphosphorylation inhibitors using docking and MD simulations [149]. Docking calculation followed by MD simulations and binding free energy analysis led to the identification of novel anti-Tau hyperphosphorylation inhibitors of Cdk5 which occupied the ATP-binding site of the enzyme [150]. A set of pyrazolopyrimidine derivatives, which are inhibitors of GSK3β, were studied with docking calculations and 3D-QSAR to develop reliable predictive models for design new compounds to inhibit Tau hyperphosphorylation [151]. Organic synthesis, molecular docking, and quantum mechanical calculations were combined with physicochemical and in vitro histochemical evaluations to identify new molecular probes for detection of Tau aggregates [152]. The interactions of positron emission tomography (PET) tracers with cryo-EM structure for the Tau fibril were defined through integrated molecular docking, MD simulations, and binding free energy calculations [153]. Experimental and theoretical docking evidence suggested that curcumin displays strong inhibition effect against Tau fibril aggregation [154]. Thermodynamic and docking binding studies suggested the formation of a hairpin structure of a Tau peptide when interacts with tannic acid, which is a key structural feature required for inhibiting Tau polymerization [155]. Pose prediction accuracy studies on GSK3β inhibitors demonstrated that cognate docking calculations successfully reproduced crystallographic poses with RMSD less than 2 Å, while cross-docking calculations produced poses that deviate from X-ray poses providing valuable insights into computational structure-based design [156]. The structural basis of targetability of monomeric Tau by small molecules was studied using molecular docking indicating the ability of small molecules, like methylene blue, to bind to monomeric Tau and influence interactions of the protein with itself and other proteins [157]. Computational pharmacophore models, molecular docking, and MD simulations were employed to identify hyperphosphorylated sites of Tau including multiple serine sites changing the flanking sequence of R1/R2 repeats, which affects binding of Tau to microtubules [158]. This docking results, allowed the identification of five ligands with high docking energies, strong hydrogen bonding and ionic interactions with the receptor, and good pharmacokinetic and physicochemical properties. To identify anti-Tau acetylation inhibitors, a computational structure-based pharmacophore model was developed using the structure of histone deacetylase 6 (HDAC6) in complex with Trichostatin A [159]. A drug-like database of 841 molecules was screened using the pharmacophore model followed by molecular docking to identify true positive inhibitors of HDAC6. The binding interactions of a novel hit compound were also studied with MD simulations revealing strong polar and van der Waals interactions with the catalytic residues of HDAC6 including His610, His611, Asp649, His651, and Tyr782. The substituent 3,4-dihydroxy contained in tolcapone and entacapone, which were used as adjunctive therapy in the treatment of Parkinson’s disease, was docked to a steric zipper structure of PHF6 (^306^VQIVYK^311^) showing close interactions of this moiety with lysine suggesting that it might be a pharmacophore for the design of Tau aggregation inhibitors [160].

The high flexibility of Tau makes it an attractive target for MD simulations, which is a very useful CADD technique for understanding the mechanism of Tau aggregation. All-atom MD simulations in explicit solvent were successful in investigating the mechanism of spontaneous Tau aggregation using PHF6 [161]. This study showed that PHF spontaneously aggregates to form multimers with β-sheet structure existing in a parallel arrangement. It was also observed that PHF6 (^306^VQIVYK^311^) monomer can be induced to form a β-sheet structure on either side of the template with the left side to be formed more favorably but not be extended, while the right side can form an extended β-sheet structure. Residues Ile308, Val309, and Try310 were reported to play an essential role during the process of β-sheet dimerization from the disordered coil structure [161]. The misfolding mechanism of the Tau monomer upon induction of formed PHFs and SFs were uncovered with a combination of conventional and steered MD simulations [162]. The results showed that the dissociation mechanisms of the boundary chain in PHF and SF differ from each other. The binding mode of a flavonoid molecule exhibiting anti-Tau aggregation properties was proposed using molecular interaction fields (MIF), pharmacophore perception, and MD simulations [163]. These results demonstrated that flavonoid ligands can induce conformational changes in the hexapeptide structure by forming pi-stacking interaction with tyrosine residue and the benzopyrone flavonoid moiety. This model was then employed to perform pharmacophore-based virtual screening on a set of compounds to identify new anti-Tau aggregation inhibitors including four tiophene derivatives which were predicted as promising Tau aggregation inhibitors [164]. Extensive replica-exchange MD simulations of Tau protein revealed dynamic conformations of K18 and K19 with some cysteine residues to be located sufficiently close to lysine residues enabling Tau self-acetylation [165]. The same study showed that β-sheet structures formed in the monomeric states provide seeds for early stage aggregation with the exposed hexapeptides PHF6* (^275^VQIINK^280^) and PHF6 (^306^VQIVYK^311^) to be the seeding nucleus. Enhanced sampling MD and conformational analysis optimized for disordered targets, coupled with computational docking and machine learning were employed to successfully identify novel chemically diverse Tau ligands including an inhibitor that delays the in vitro aggregation reaction without affecting the amount of aggregate formed at the steady state [166]. Other CADD studies for the development of anti-Tau inhibitors are summarized in previously published review articles [167,168].

## 4. Future Perspectives

Alzheimer’s disease has unmet medical needs especially for disease modifying therapies. The role of β amyloid in the pathogenesis of AD has been central to the drug discovery efforts for many years, and has led to a substantial number of drugs targeting the β amyloid (β and γ secretase inhibitors, both of which reduce the amount of β amyloid formed, immunotherapy to clear β amyloid, and aggregation inhibitors) which however most of them have failed in clinical trials. In fact, between 1995 to 2014 the industry invested in 1120 pipeline drugs for AD both disease-modifying and symptomatic therapies. Despite this effort, the four currently marketed drugs were approved a decade ago and are symptomatic [169]. No disease-modifying therapies have succeeded so far, with a number of high-profile late-stage failures targeting Aβ including β secretase (BACE), semagacestat [170], bapineuzumab [171], and solanezumab [172], while anti tau drugs have only recently been tested. As a result, the overall success rate for AD is only 0.5% compared to 4.1 which is the industry average [169].

The reasons for the relatively high failure rates are being actively sought, and many explanations have been proposed. Some of these include the correct dose given, the correct form of Aβ as a target, and the appropriateness of the clinical trial. Regarding the later, since most of the drugs are disease modifying the length of the clinical trial seems to be critical to detect such changes. However, sometimes it is not possible to extent the length of the trial to see an effect as this will require more subjects and sites, at the expense of loosing subjects, and life events may undermine the clinical trial. In addition, there are issues with inaccuracy with clinical trials that are dependent on using clinical ratings as outcome measures. In this respect, there may be issues with ratings and the raters many be inexperienced, and this may compromise the CT and lead to failure of the drug, rather than the drug itself. Similarly, measurement errors and a lack of specificity during diagnostic evaluations and qualifications of subjects for clinical trials eligibility can lead to subjects being incapable of responding to treatment due to misdiagnosis, genetics, or specific pathology. Of particular concern is the possibility that some enrolled patients are already in advanced stages with little hope for a disease modifying effect with any drug [173,174]. Another possibility includes the variability of measurement of endpoints within or between the individuals based on the clinical endpoints assessed. This variability in measurements implies that it will always be difficult to detect even a good treatment efficacy in a trial of 5 year’s duration. The complexity of the AD pathophysiology is another factor [175]. Despite the fact that many drugs have failed, some make a comeback like adabanumab (a β amyloid targeting drug) that after initial failure it seems that after further analysis of the data there is hope that it could prove efficacious [176]. 

The last few years new evidence suggest that Tau protein is also important. Several studies have shown that Aβ and phosphorylated Tau (p-Tau) independently predict disease progression [177], and a hypothetical framework proposes that both proteinopathies synergistically potentiate downstream neurodegeneration [178]. The presence of such a synergism would suggest that the effect of Aβ and p-Tau on the progression of AD taken together is greater than the sum of their separate effects at the same level. Indeed, recent findings support that progression of AD dementia is driven by the synergistic rather than a mere additive effect between Aβ and p-Tau proteins [179] changing the way treatment may be directed.

Indicative of the importance of amyloid-β and p-Tau in AD is the efforts in drug development targeting these two proteins. Currently, there are 132 agents in clinical trials for the treatment of AD of which 96 (73%) are disease modifying. Out of these, 38 (40%) have amyloid as the primary target (18 are small molecules and 20 are monoclonal antibodies or biological therapies) and 17 (18%) of the 96 drugs have Tau as a primary or combination target (7 are small molecules and 10 biologics). Amyloid is the most common specific target in phase 3 and phase 2 disease modification trials [13]. More precisely, in 2019 there were nine drugs targeting Aβ in phase III of which one was aggregation inhibitor, two BACE, and six immunotherapies, while one was a dual anti amyloid anti Tau. In phase II, there were two drugs targeting both amyloid and Tau, eight were anti Tau and 18 were targeting Aβ [13]. In contrast, in 2016 there were 13 drugs targeting Aβ in phase III of which four were BACE inhibitors, five immunotherapies, and one was an anti Tau. In the same year, in phase II there was one anti Tau and 12 targeting Aβ [180]. As it is evident, and as expected from the scientific findings a shift was made to Tau as well as inhibitors targeting both Tau and Aβ. Considering the speculative reasons for the failures, as well as the new drugs in the pipeline and the insight gained from all the failures regarding toxicity and efficacy, both Aβ and Tau are still relevant targets, and targeting both is likely to have a better efficacy.

It has long been suggested and it is actually more intensively studied nowadays that for complex neurological diseases like AD, multi target drug therapy is probably a better solution rather than focusing on a single target. Given the variety of factors associated with the onset, progress and severity of AD, increasing their degree of pathophysiological complexity, and associated to the inefficiency of the current therapeutic arsenal available, it becomes unavoidable to adopt a new concept for the rational design of new drugs against AD. The new findings on the requirement of both Tau and amyloid on this comes to strengthen this notion for AD. As such, drug candidate prototypes with dual mode of action were the first attempts to look up ligands recognized by more than one molecular target, or more than one site on the same macromolecular target. These molecular entities could lead to the identification of new bioactive chemical entities with selective affinity for multiple targets, preferably in different biochemical cascades. Therefore, these innovative ligands could play a significant role in the advance of a broadly and more efficient therapy, and perhaps, in the cure of AD [181].

Taking into consideration recent findings that that Aβ and Tau proteins work synergistically and that increased β amyloid levels means increased Tau levels [182], there is a possibility that a properly selected dual inhibitor would be efficacious and have true disease modifying effects in the clinic. Whether targeting both proteins is better than a single, has not been tested yet, however all the evidence points to this direction. Regarding these multi target drug ligands (MTDLs) several efforts have been made primarily on the acetyl cholinesterase inhibitors (Achei) that also possess amyloid inhibitory effects due to their ability to inhibit the peripheral anion site of Acetycholinesterase (Ache) thus assuming β amyloid anti aggregation effects [183,184]. One such drug is the marketed symptomatic drug donepezil while many others have also been synthesized. Despite this the disease modifying effects of such drugs have not been proved. Some other attempts have been made to design dual inhibitors targeting β amyloid and Tau. One such attempt is a derivative of curcumin that acts both as Tau an β amyloid aggregation inhibitor [185] and a second molecule also targets Tau hypephosphorylation and β amyloid aggregation [186]. Despite the fact that most efforts to date have focused on the β amyloid hypothesis, Tau as a target is becoming a strong player. Therefore, despite the failures, considering that AD is a complex disease, β amyloid is still an attractive target, and given the recent findings that Tau synergizes with Aβ, drugs targeting both (as MTDL) may prove more efficacious. With the increased number of drugs targeting Tau that are in clinical trials now compared to a few years ago it gives promise that in the near future either the MTDL, or single anti-Tau or anti-amyloid will be effective. Finally, CADD gives unique opportunity to design and develop drugs acting as single or dual inhibitors on these important targets. This is especially true in designing dual small molecule inhibitors where the binding characteristics of the targets have to be delineated and meticulously detailed in order to make better use of this information to design more efficacious drugs. This review article highlights these characteristics for future use.

## 5. Conclusions

Alzheimer’s is a neurodegenerative disease characterized by progressive neuronal death/loss and synapses loss in human brain. The amyloid-β and Tau pathways leading to amyloid plaques and neurofibrillary tangles, respectively, are the main cellular pathways for the development of anti-Alzheimer’s therapies. The structure and functions of the druggable targets involved in these two pathways are discussed in this review article. Examples of various computer-aided drug design strategies presently employed for the development of small molecule inhibitors are also reported. These examples mainly involve the application of computational structure-based design and ligand-based design including molecular docking, molecular dynamics simulations, quantitative structure-activity relationships, fragment-based design, and pharmacophore based design for the development of small molecule inhibitors. The main biological targets discussed herein include β-secretase, γ-secretase, and Tau as well as kinases and histone deacetylase 6 which are involved in hyperphosphorylation and acetylation of Tau, respectively. Finally, examples of computational approaches used for the development of anti-amyloid and anti-Tau aggregation inhibitors are also summarized.

## Abbreviations

AD	Alzheimer’s disease;
CNS	Central nervous system
Aβ	Amyloid-β;
Tau	Tubulin-associated unit
NFTs	Neurofibrillary tangles
CADD	Computer-aided drug design
QSAR	Quantitative structure-activity relationship
APP	Amyloid precursor protein
Aβ_40_	Amyloid-β peptide consisting of 40 residues
Aβ_42_	Amyloid-β peptide consisting of 42 residues
BACE1	APP cleaving enzyme I
BACE2	APP cleaving enzyme II
TGN	Trans-Golgi network
MM-GBSA	Molecular mechanics generalized Born surface area
MD	Molecular dynamics
SOMFA	Self-organizing molecular field analysis
QM/MM	Quantum mechanics/molecular mechanics
i-CLiP	Intramembrane cleaving proteases
PS	Presenilin
NCT	Nicastrin
APH-1	Anterior pharynx defective 1
PEN- 2	Presenilin enhancer 2
NTF	N-terminal fragment
CTF	C-terminal fragment
GSIs	γ-secretase inhibitors
GSMs	γ-secretase modulators
Aβ_49_	Amyloid-β peptide consisting of 49 residues
Aβ_46_	Amyloid-β peptide consisting of 46 residues
Aβ_43_	Amyloid-β peptide consisting of 43 residues
HQSAR	Hologram quantitative structure-activity relationship
PHFs	Paired helical filaments
SFs	Straight filaments;
GSK-3β	Glycogen synthase kinase-3β
Cdk5	Cyclin-dependent kinase 5
CK1	Casein kinase 1
PKA	Protein kinase A
PP2A	Protein phosphatase 2
microED	Micro-electron diffraction
Cryo-EM	Cryo-electron microscopy
PET	Interactions of positron emission tomography
RMSD	Root mean square deviation
HDAC6	Histone deacetylase 6
MIF	Molecular interaction fields.
